# Extraordinary Sex Ratios: Cultural Effects on Ecological Consequences

**DOI:** 10.1371/journal.pone.0043364

**Published:** 2012-08-27

**Authors:** Ferenc Molnár, Thomas Caraco, Gyorgy Korniss

**Affiliations:** 1 Department of Physics, Applied Physics, and Astronomy, Rensselaer Polytechnic Institute, Troy, New York, United States of America; 2 Department of Biological Sciences, University at Albany, Albany, New York, United States of America; University of Alberta, Canada

## Abstract

We model sex-structured population dynamics to analyze pairwise competition between groups differing both genetically and culturally. A sex-ratio allele is expressed in the heterogametic sex only, so that assumptions of Fisher’s analysis do not apply. Sex-ratio evolution drives cultural evolution of a group-associated trait governing mortality in the homogametic sex. The two-sex dynamics under resource limitation induces a strong Allee effect that depends on both sex ratio and cultural trait values. We describe the resulting threshold, separating extinction from positive growth, as a function of female and male densities. When initial conditions avoid extinction due to the Allee effect, different sex ratios cannot coexist; in our model, greater female allocation always invades and excludes a lesser allocation. But the culturally transmitted trait interacts with the sex ratio to determine the ecological consequences of successful invasion. The invading female allocation may permit population persistence at self-regulated equilibrium. For this case, the resident culture may be excluded, or may coexist with the invader culture. That is, a single sex-ratio allele in females and a cultural dimorphism in male mortality can persist; a low-mortality resident trait is maintained by father-to-son cultural transmission. Otherwise, the successfully invading female allocation excludes the resident allele and culture and then drives the population to extinction via a shortage of males. Finally, we show that the results obtained under homogeneous mixing hold, with caveats, in a spatially explicit model with local mating and diffusive dispersal in both sexes.

## Introduction

Since Fisher’s [1] classic insight, sex-ratio evolution [2]–[4] and the impact of a given sex ratio on ecological dynamics [5]–[8] have remained central issues in population biology. Fisher [1] noted that neither sex should be rarer at evolutionary equilibrium, a consequence of frequency-dependent selection. That is, equal investment of reproductive effort in the two sexes – commonly implying a sex ratio close to unity – can be evolutionarily stable [9].

Hamilton [10] studied sex ratios departing significantly from unity, emphasizing that Fisher’s argument does not apply when a sex-linked gene controls sex ratio at birth. In particular, if a gene governing sex ratio occurs in the heterogametic sex only (females in the 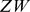 system, and males in the 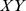 system), the gene’s fitness depends only on the number of heterogametic offspring produced. The frequency of such a gene may advance rapidly, endangering population persistence [11], [12]. That is, a biased sex ratio can leave members of the more common sex without mates; the consequent “marriage squeeze” [5] may lead to population decline [10], [13]. Equivalently, an Allee effect (dependent on the density of each sex) can limit the degree of sex-ratio bias, for given total density, capable of averting direct decline to extinction [6], [14]–[16]. Our study supposes that an extraordinary sex ratio’s ecological consequence, population persistence or extinction, depends on interaction with a culturally inherited trait.

Cultural traits may enforce a between-sex mortality difference [17]. In certain human cultures, infanticide and neglect increase female mortality [18], [19]; Laland *et al*. [20] assume that these cultural traits are transmitted vertically, *i.e*., parent to offspring. In other species, vertical cultural transmission clearly causes between-sex differences in habitat choice, tool use or foraging behavior, but their relationships to sex-specific mortality rates are unknown [21]–[24]. Our models explore how a cultural trait influencing male mortality might govern the ecological consequences of sex-ratio evolution. We treat sex ratio as a sex-linked genetic trait, and restrict cultural transmission to the vertical case [25]. Our two-sex population dynamics assumes competition for a growth-limiting resource; competition generates a strong Allee effect. Within a group, each female carries the same sex-ratio allele, and each male experiences the same mortality rate; parameters differ between groups. Resource competition is preemptive; each group has the same niche [26]–[28].

Our approach assumes pairwise competition between resident and invader groups, where group refers to population structure, not the level of selection. In Sober’s [29] terminology, we associate properties driving selection with groups, and associate the objects of selection with individuals – individual females in this case. The resident group (sex ratio, male mortality culture) rests at ecological equilibrium, and we ask if a rare, different group can invade the resident. Our results for invasion, extinction and (cultural) coexistence indicate how resource competition, cultural variation and sex-ratio evolution interact. Ecological invasion often has a distinctly spatial character [30], [31]. Therefore, we extend our model beyond the assumption of homogeneous mixing, and introduce spatial detail by analyzing the model’s reaction-diffusion extension.

## Methods

### General Assumptions

In birds (and butterflies) sex determination follows the 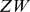 system. 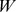 is the sex-determining chromosome; females are 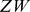, and males are 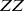 [32]. Our model assumes that the 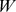 chromosome carries an allele fixing the sex ratio among that female’s offspring. The sex linkage means that a female inherits her mother’s sex ratio, and the sex-ratio gene never occurs in males. Hence, the fitness of the sex-ratio allele (of any gene on the 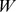 chromosome) is advanced only through production of daughters [10]. To focus our discussion accordingly, we model the “female ratio,” the proportion of a female’s offspring born female. Females of a single group carry the same sex-ratio allele.

The assumption of sex-linkage might seem restrictive. However, in a number of bird species, individual females shed Z-chromosome and W-chromosome bearing eggs non-randomly [33], [34]. The observed variation in sex ratio among females may reflect facultative plasticity [35], but could generate some of the population-dynamic consequences of sex ratio that we model.

All members of a given group share a vertically transmitted cultural norm that governs male behavior which, in turn, fixes the male mortality rate for that group. Females of different groups share the same mortality rate. Hence, for simplicity, we assume a female adopts her mother’s culture. If both parents belong to the same group, their son faithfully acquires the parental culture. When parents of different cultures (groups) mate, a son acquires one or the other culture, each with probability 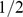.

To address competition between groups, we envision a resident group (a single female ratio and a single male mortality rate) at ecological equilibrium in a resource-limited environment. We then introduce (*via* demic/genetic migration) a small inoculum of an invader group. The resident and the rare invader differ in female ratio and ordinarily differ in male mortality. The competitive dynamics proceeds to ecological equilibrium. If the rare female-ratio allele has positive growth, it will drive change in culture. Since individuals mate randomly, extinction of a group’s female-ratio allele need not always imply loss of the associated cultural trait. However, loss of a cultural mortality trait implies that the associated female-ratio allele has been excluded competitively.

Our population dynamics differs from models for gene-culture coevolution where different alleles and cultural traits directly affect each other’s evolution [20]. Our model’s cultural trait directly influences the resident’s population density and the invader’s growth rate when rare; female ratios and male mortalities interactively drive the invader’s dynamics. We do not assume functional dependence between the genetic and cultural traits. Rather, we evaluate consequences of the feasible range of male-mortality rate combinations for the entire range of female ratio combinations (resident and invader).

### Mathematical Model

Consider two-sex population growth with two female ratio/male mortality groups; the groups allow us to model resident-invader differences. When a female of group 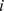 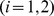 reproduces, the resulting offspring is female with probability 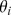, and male with probability 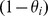, independently of the group of the male with whom she mates. 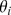 is the female ratio for group 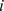, transmitted faithfully from mother to daughter. Different groups, by definition, differ in female ratio. All females have the same mortality rate, 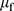.

A male’s group specifies his mortality rate, 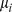 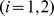. If male mortality exceeds the rate for females, 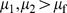. But we do not exclude the case where the female mortality exceeds one or both male rates. If both parents belong to the same group, each male offspring has that group’s mortality rate, acquired by vertical cultural transmission. If a male’s parents belong to different groups, the male acquires mortality rate 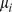 with probability 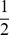.


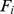
 and 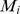 represent the global density of females and males, respectively, of group 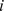. All individuals require the same resources, so that population growth at larger densities will self-regulate. The preceding assumptions imply the following dynamics under homogeneous mixing (or “mean-field”):
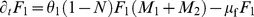


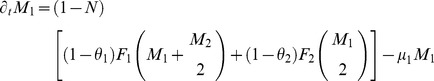


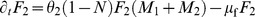


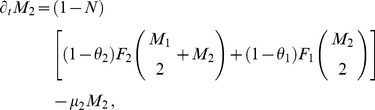
(1)where 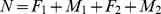 is total global density; 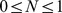. Males encounter females as a mass-action process, modeling random mating [14], [25]; more complicated assumptions about pair formation suggest different “marriage functions” [8]. The fraction of matings that reproduce successfully equals the unoccupied fraction of the environment, 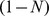. Below we take group 1 as the resident, and identify group 2 as the (initially rare) invader.

If only a single group occupies the environment, the equations reduce to those studied by Tainaka et al. [12]:
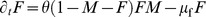


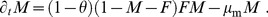
(2)


The authors focused on the symmetric case, 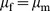. An important feature of this model is that the cubic dynamics produces a strong Allee effect [15], [16]. That is, there exists a threshold for the initial population density, below which growth is necessarily negative, and extinction must follow [14]. The single-group model serves as the starting point of our analysis. In particular, initial conditions of our competition dynamics will depend on the stable, non-trivial fixed point of the single-group model (corresponding to positive equilibrium densities for females and males of group 1).

### Analytic and Numerical Methods

We assume that the population dynamics is fast compared to the time scale of immigration (invasion of new gene-culture groups). Then female ratio should evolve through a series of successful invasions of populations resting at demographic equilibrium. Therefore, we obtained the fixed points (stationary solutions) of Eqs. (1) analytically (see File S1), and we used numerical integration to analyze their local stability.

Since this study employs extensive numerical integration, we justify our choice of an ordinary differential equation (ODE) solver. Equations (1) are strongly coupled and may become stiff, a challenge to the solver. Speed is another important factor because we mapped the entire parameter space of the model, which requires a very large amount of computation. We chose the explicit fourth-order Runge-Kutta method [36], which gives the precision we require. We utilized adaptive time stepping to avoid problems with any potential stiffness, and to increase integration speed when the slopes of the densities were small. Since we are interested in stationary solutions of the equations, the stopping condition for the integration specifies that all numerical derivatives are smaller than a predetermined limit:
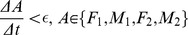
(3)In our ODE numerical integrations, we set the stopping condition at 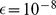.

Eqs. (1) assume that each individual encounters any potential mate at the same average rate. But full mixing will seldom prove realistic, since mating encounters ordinarily occur more frequently between nearby, than between distant pairs. Spatially structured mating can be especially important during ecological invasion, because introduced invaders often cluster locally [30], [31], [37]–[39]. To address spatial detail, we generalized Eqs. (1) as a reaction-diffusion system [40]. To model spatially structured mating encounters, we replaced the homogeneous global densities with the corresponding local densities (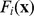, 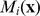) at location 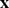. To model dispersal we added a diffusion term (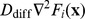 and 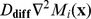 for group 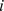) to the respective equation of motion. To integrate the spatial model numerically, we discretized the partial differential equations (PDEs) to ODE equations (based on the Method of Lines technique [41]) on a rectangular grid of size 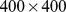 (representing an area of 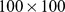 units), using Neumann boundary conditions. We integrated the resulting ODEs using an explicit Euler time stepping, for which we chose a sufficiently small time step (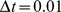). These parameters allow us to use diffusion coefficients as large as 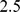 without producing finite-size effects, or instability. For the spatial model, we defined global equilibria with the stopping condition 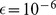.

## Results

### Stability of the Resident

Before we address the dynamics of competitive invasion, we must review [12] and establish conditions for an ecologically stable resident population. A stable resident occupies the habitat alone, at a real, positive fixed point where self-regulation limits growth, governed by Eqs. (2). In general, the system has three fixed points: the trivial solution at zero density, and a pair of nonzero fixed points. Extinction is always stable; one of the nonzero fixed points is unstable, and the other one is stable. The nonzero fixed points, hence a stable positive equilibrium, exist if (as shown in File S1)
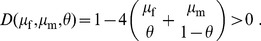
(4)


**Figure 1:**
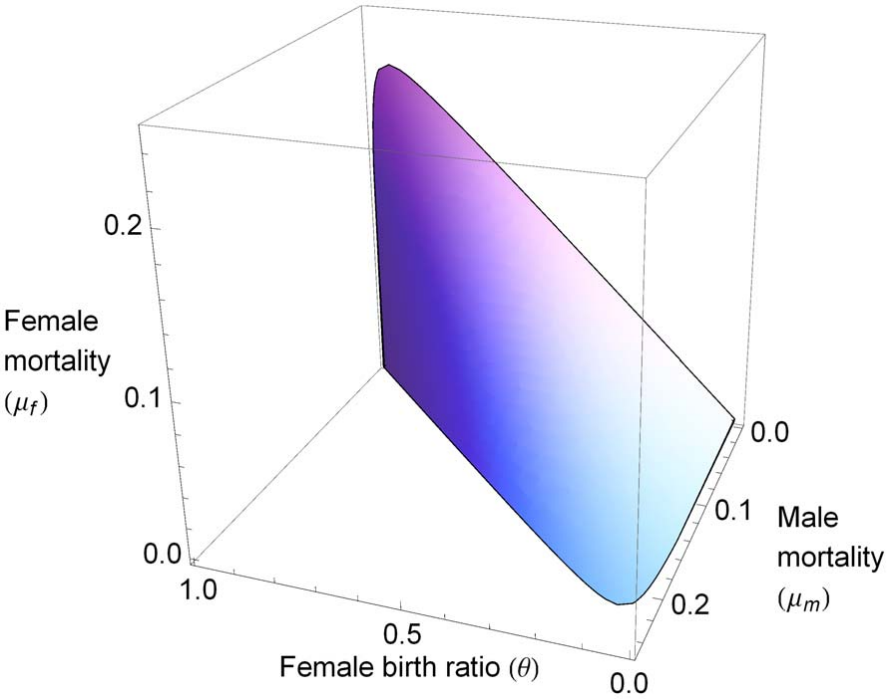
Region of the parameter space where the resident is persistent. Parameter space region defined by Expression (4). Choosing parameters from the indicated domain always results in a stable nonzero population, given sufficiently high initial densities.

The necessary condition for this inequality is
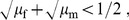
(5)in which case there exists a female-ratio continuum, 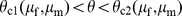, where the population might persist. “Might persist” means that a positive equilibrium exists, and initial conditions determine whether or not the positive equilibrium attracts the dynamics. If expression (4) fails to hold, the system exhibits only the trivial fixed point, stable extinction. A resident population’s persistence, then, depends on interaction of the female ratio at birth with the sex-specific mortality rates. In particular, when expression (4) holds, any increase in the culturally transmitted mortality trait 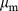 shrinks the range of female ratios maintaining an extant resident population (see File S1). More generally, Figure 1 depicts the region of the parameter space satisfying expression (4).

We performed a linear stability analysis of the system, using Mathematica [42]. The results show that if condition (4) is met, then the larger (“
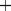
”) roots in Eqs. (S6) (provided in File S1) are always locally stable, and the smaller roots are always unstable.

We present analytical formulae for the stable stationary densities in File S1. We used those formulae to quantify our numerical integration’s accuracy. We performed 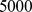 test runs with randomly chosen parameters that obey Expression (4). For 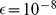, we find that the absolute difference of the numerically computed fixed point was only 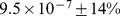 from the analytical value, with 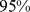 confidence. This accuracy suffices for our work.

To reach stable, positive equilibrium, population growth must overcome a strong Allee effect [16], which defines a separatrix on the phase map of initial female and male densities. Below the separatrix extinction always results, independently of other parameters, since growth is negative. Above the separatrix the population grows to self-regulated equilibrium. To find this threshold numerically, we select model parameters and fix the initial female density. Then we conduct a binary search for the initial male-density threshold value, numerically integrating Eqs. (2) until they converge to a stationary value (zero or nonzero). Using this method we can determine the threshold value with arbitrary precision.

**Figure 2:**
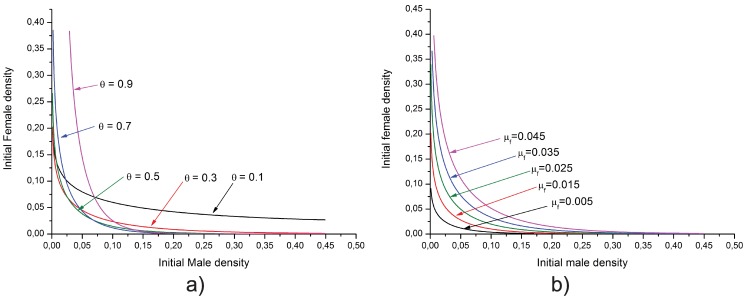
Allee threshold of the resident. Survival/extinction threshold defined by the Allee-effect, at various female ratios (a) and various female mortalities (b). Other parameters: (a): 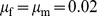; (b): 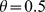, 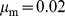.

Figure 2 displays the Allee-threshold for various parameter combinations. In Figure 2(a), where 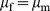, an unbiased female ratio 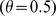 allows the lowest total population density before extinction due to the Allee effect ensues. When the sexes have the same mortality, unbiased sex allocation also maximizes total population density at positive equilibrium [12].

Figure 2(b) verifies that increasing female mortality, 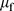, for given 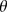 and 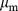, expands the region where the Allee effect leads to extinction. Not surprisingly, increasing male mortality produces a parallel effect. Mortality-rate asymmetry and biased female ratios distort the shape of the thresholds in Figure 2, but the same general patterns emerge.

For a resident population, we have specified how existence of a positive equilibrium depends on the interaction of female ratio at birth and sex-specific mortalities. We also have shown that initial conditions (given existence of a positive equilibrium) required to avert extinction due to the Allee effect depend on the same parameters. A practical consequence is that we must choose initial densities for numerical integration carefully, so that when the competitive dynamics results in extinction, we can clearly identify the reason as either the Allee effect or exclusion.

### Ecological Competition: Female Ratio and Invasion

To quantify how population consequences of female-ratio evolution can be affected by male mortality, we must have an ecological understanding of the two-group competition model, [Eqs. (1)]. The system has nine fixed points; see File S1. One is the trivial fixed point where all densities vanish. We can easily identify four more fixed points related to those of the single-class case; there are two symmetric pairs. At these fixed points, competitive exclusion leaves one group extinct, and one extant. Exclusion implies that one group’s female ratio allele and its male-mortality cultural trait have both gone extinct. Only one of these four, non-trivial fixed points is locally stable: the “
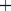
” solution [Eq. (S11)] of the group with the *greater female ratio*. Assuming that 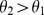, a necessary condition for this fixed point’s local stability is 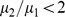 (see File S1 for details). We shall refer to a fixed point where one allele/culture persists after excluding the other as a type-I fixed point. When male mortality rates imply a type-I fixed point, the greater female ratio always excludes the lesser ratio.

The four remaining fixed points (again, forming two pairs by symmetry) are qualitatively distinct from those discussed above. At these fixed points only one female-ratio allele remains extant, but male mortality traits “coexist.” That is, the population is genetically uniform, in that all females carry the same female ratio allele. But the (male) population is culturally dimorphic; father to son transmission [see Eqs. (1)] maintains the culture of the group whose females have been excluded competitively. Consider a stable fixed point of this sort, when 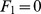. The necessary conditions are 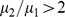, 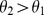, and positivity of the discriminant
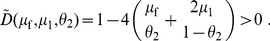
(6)


**Figure 3:**
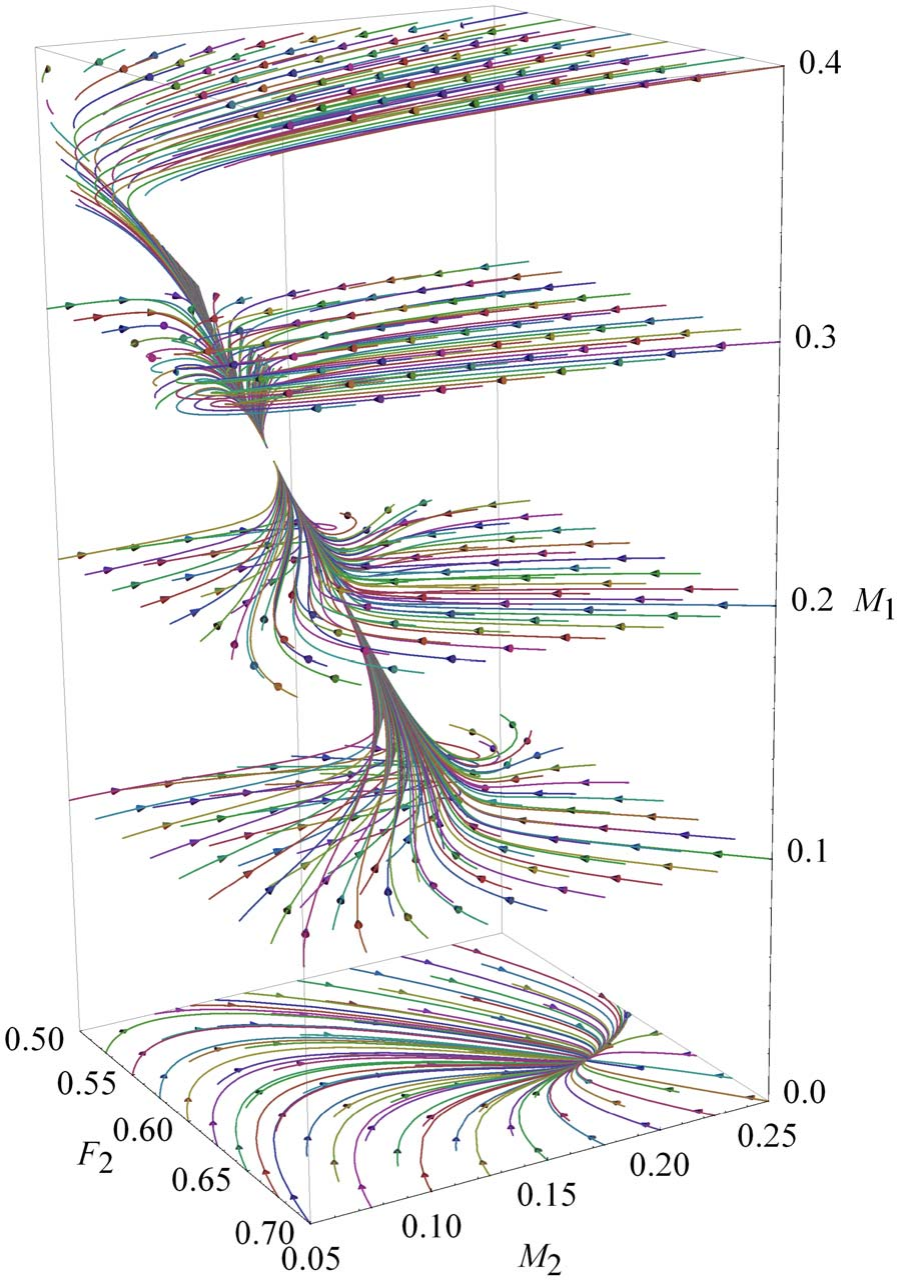
Mean-field density flows. Density flows in the 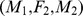 space (restricted to 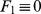) with type-I (“saddle”, 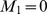) and type-II (“stable”, 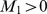) fixed points for 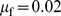, 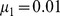, 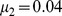; 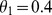, 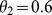.

The preceding condition holds if
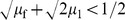
(7)and 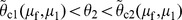. For mathematical details, see File S1. We refer to stable fixed points combining a single female ratio and a male cultural dimorphism as type-II fixed points. Summarily, the model does not permit equilibrium coexistence of female ratio alleles, but can permit equilibrium diversity in cultural traits governing male mortality. Also note, as is clear from the above conditions, that of type-I and type-II fixed points *only one* can be stable at a time. In Figure 3 we illustrate the flow in the mean-field dynamics for a set of parameters when both type-I and type-II fixed points exits, but in the presence of co-occurring males of the other allele, only type-II is stable.

Having obtained the nine fixed points for the two-group model analytically, we approached the stability analysis numerically. Analytical study of the system’s stability proves difficult, due to the number of variables and parameters (4 variables and 5 parameters). To be as thorough as possible, we performed numerical integration systematically to span a significant region of the five-dimensional parameter space. The range and step of the parameters in our numerical scheme can be found in Table 1. Each run begins with a stationary resident population, with allele 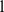 and cultural trait 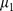. If model parameters allowed a stable positive equilibrium, we chose initial densities accordingly. We then introduce the invaders, with female-ratio allele 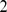 and cultural trait 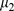. For each set of parameters (in each series) we performed two runs, one with infinitesimal initial density of invaders (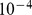) and one with high invader density (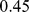).

**Table 1:** Parameter regions and step sizes for numerical integration.

Parameter	Lower bound	Upper bound	Step
Series 1			
*θ*_1_	0.01	0.99	0.01
*θ*_2_	0.01	0.99	0.01
*μ*_1_	0.01	0.04	0.005
*μ*_2_	0.01	0.04	0.005
*μ_f_*	0.01	0.04	0.005
Series 2			
*θ*_1_	0.1	0.9	0.1
*θ*_2_	0.1	0.9	0.1
*μ*_1_	0.001	0.1	0.001
*μ*_2_	0.001	0.1	0.001
*μ_f_*	0.001	0.04	0.01

Each set of parameters identifies two runs: one with high (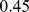) and one with low (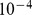) initial invader density.

**Figure 4:**
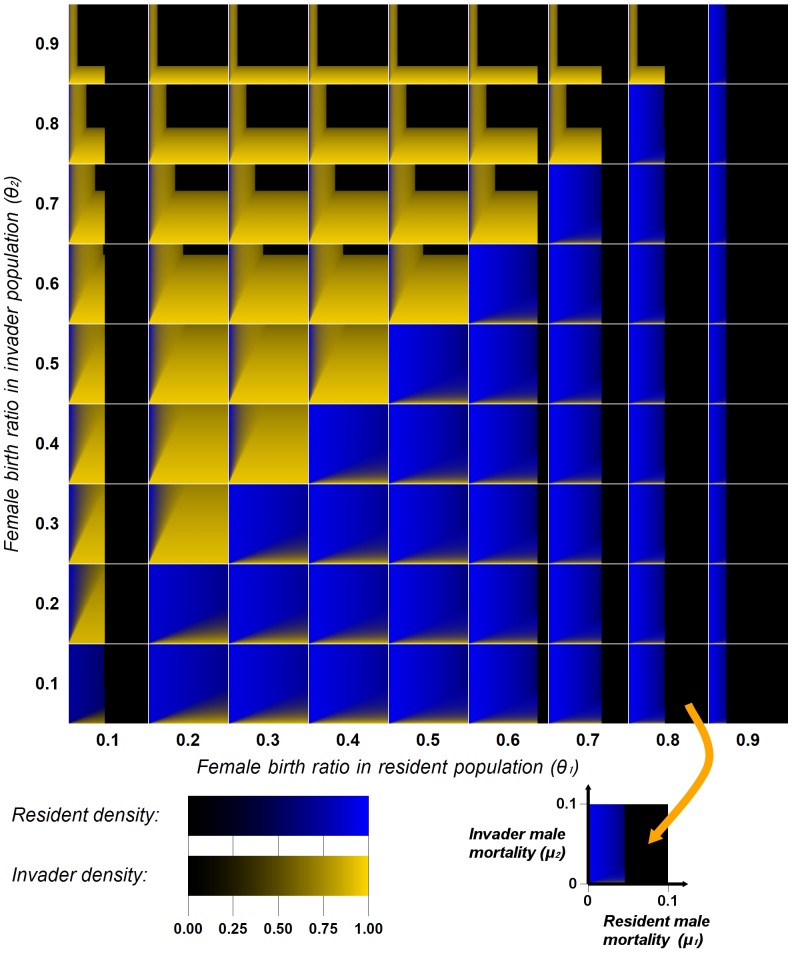
Stationary population densities. Numerical integrations are performed for the scenario where the persistent stationary resident (group 1) is invaded by group 2, initially at an infinitesimal density (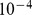). Large axes indicate common parameters in rows and columns; every tile has the same axes, scaled as indicated in the bottom right corner. Color scales use independent color channels, therefore, resident and invader densities are shown independently. Female mortality is fixed: 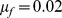.

To portray the results, we generated a number of “4D” plots. Each shows a table containing 2D plots with the results of each run; the axes of each 2D plot are values of the same two cultural parameters (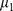 and 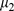, all with the same range). Another two parameters (female ratios 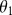 and 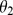) vary across the rows and columns of the tables (the 4D plots). We produced as many tables as required by the range of the fifth parameter (female mortality 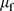). In each 2D plot, one pixel represents the final stationary densities of the female ratio alleles. The pixel’s location corresponds to the parameters for which it was computed; resident and invader allele densities are shown on different color channels. This way, we can visually compare all the results simultaneously, simplifying the analysis greatly. Figure 4 shows one 4D plot; the associated parameter ranges produce the full set of the model’s outcomes.

In what follows, we investigate the necessary and sufficient conditions for successful (pairwise) genetic invasion of the resident female ratio, and the necessary conditions for cultural “coexistence”.

#### Invasion and exclusion

Our numerical results reveal immediately that female ratios determine the outcome of invasion; a successful invader in pairwise competition has the greater female ratio. That is, successful invasion always requires 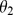 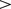 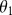, and 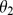 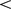 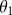 assures that the resident resists invasion. When the invader has the greater female ratio, it excludes the resident allele competitively. Furthermore, successful invasion by a female-ratio allele assures that the associated cultural trait (with value 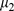) advances from rarity. As a numerical check, we note that both infinitesimal and high invader densities always result in identical final densities.

Since the female-ratio allele is sex-linked, dependence of invasion on 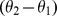 simply recalls Hamilton [10]. But in our model, the ecological effect of invasion depends on the culturally transmitted trait. Suppose that successful invasion excludes both the resident female ratio allele (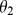 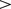 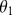) and the resident cultural trait (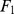 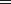 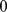, 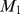 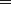 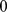). From File S1, the necessary conditions for invasion and combined genetic/cultural exclusion (type-I fixed point) are:
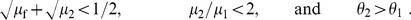
(8)


Sufficient conditions for invasion and exclusion of both resident traits further require: 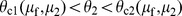, ensuring that the invader attains positive stable equilibrium.

**Figure 5:**
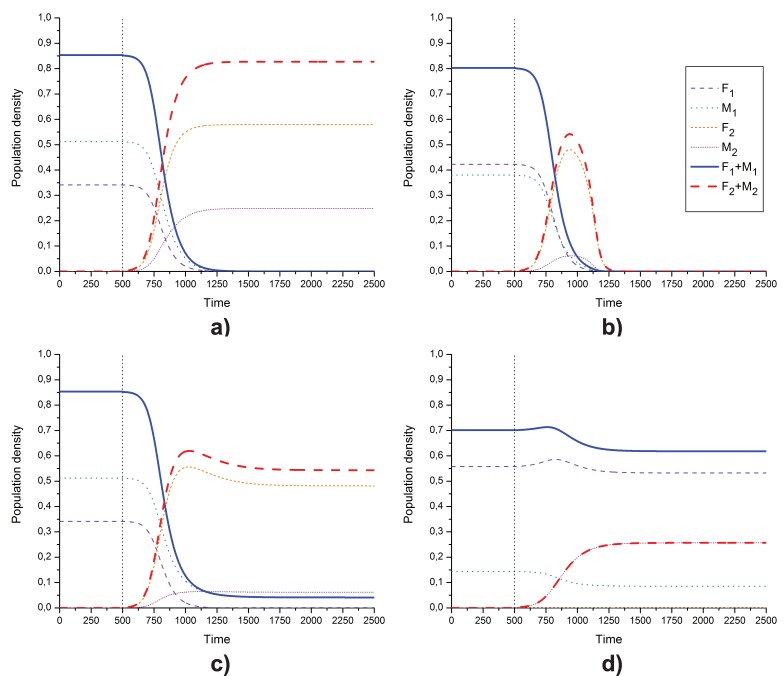
Population-density time series. Panel (a) shows successful invasion; (b) shows invasion followed by extinction; (c) shows coexistence of resident males with the invader allele; (d) shows coexistence of invader males with resident allele. The vertical dotted line indicates the time when the invader was added to the system, at 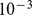 density (both males and females). Legends shown to the right of panel (b) describe data on all four panels. Common parameter: 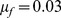. Individual parameters: (a) 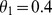, 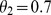, 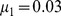, 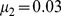; (b) 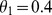, 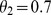, 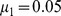, 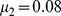; (c) 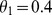, 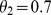, 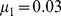, 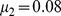 (here, 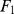 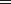 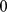 in the final equilibrium); (d) 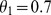, 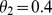, 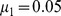, 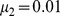 (here, 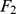 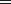 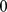 in the final equilibrium).

Figure 5(a) shows an example of successful invasion leading to exclusion of both the resident allele and resident culture. Following introduction of the invading group, the resident density drops quickly, and the successful allele (females) and successful culture (observed in males) advance to become the new resident group. Invasion, full exclusion of the resident, and population persistence first require that the successful invader’s male mortality assures, given female mortality 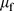, feasibility of a stable, positive equilibrium in the absence of between-group competition. Expression (S7) gives the explicit cultural constraint on female ratios guaranteeing a stable, positive equilibrium. Assuming this condition holds, the invader must, secondly, have the greater female ratio. But the invader’s demographic advantage of a greater female ratio will not exclude both the resident allele and resident culture unless constraints on the mortality rate are satisfied. Specifically, the invader’s cultural trait 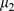 cannot exceed either 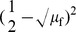 nor 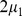.

#### Invasion and cultural coexistence

Recall that Eqs. (1) do not have fixed points where differing female ratios co-occur. The model, however, does allow for cultural coexistence, where males of both groups co-occur, but females of only one group remain extant. For details, see File S1.

In one such scenario, resident females are excluded (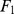 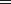 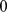), but resident males, a cultural designation, persist (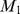 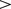 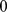). Necessary conditions for this type of coexistence (*i.e*., for a type-II stable fixed point) are
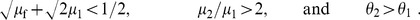
(9)


For sufficiency, the invaders’ female ratio must fall into a finite interval, 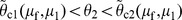, given by the positivity requirement of the corresponding discriminant [Eq. (6)].

Figure 5(c) displays an example where the resident culture, but not the resident allele, persists after successful invasion. The invader has the greater female ratio, and excludes the resident allele competitively. The final equilibrium state is a type-II fixed point where the resident’s male-mortality trait persists via father-to-son cultural transmission. The ratio of males at dynamic equilibrium is 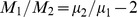. Note that the competitively driven increase in female ratio produces a decrease in total population density (females plus males) at equilibrium [Figure 5(c)].

By the symmetry of the equations, there also exists a type-II stable fixed point with 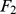 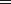 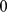. That is, the resident population resists invading females, but the introduced cultural trait advances from rarity. Put simply, we can exchange the resident-invader roles of the two groups, and reach the same dynamic equilibrium. Necessary conditions for this case are
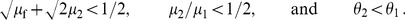
(10)


Here, the introduced female ratio 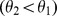 is repelled. However, the invading male mortality culture, introduced at infinitesimal density, advances and persists at equilibrium; see Figure 5(d). The ratio of males at this equilibrium 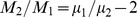.

Figure 4 includes cases of equilibrium cultural coexistence. For example, condition (9) is visible in tiles where 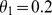 and 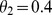; the sharp change in color along the line 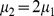 indicates the condition for cultural coexistence. When this condition is not satisfied, the culture associated with the lower female birth ratio always declines to extinction. In both cases, the fixed points found numerically are identical to the analytical fixed points for the respective equilibria: Eqs. (S16) for cultural coexistence, and Eq. (S11) for competitive exclusion of both allele and culture.

#### Invasion to extinction

Given the competitive advantage of increased female allocation in our model, evolution of the sex-linked trait might threaten population persistence. Our model’s dynamics includes a case where successful invasion of a stable resident is followed by extinction of the entire population. We observe this result in numerical experiments where the invader has both the greater female ratio and the greater male mortality rate, so that Expression (4) fails to hold. The greater female ratio drives invasion, but the invader’s combined genetic-cultural demography does not satisfy the condition for a stable, positive equilibrium. Hence, the successful invader would not advance from rarity absent the resident group.

Figure 4 shows an example of invasion to extinction; note the black region of the tile where 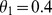 and 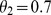. For a particular mortality-rate combination, Figure 5(b) depicts the time-dependent densities for a case of invasion to extinction. The necessary conditions for invasion, see Eq. (8), are met. However, 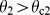. Hence the invader grows when rare and excludes the resident, but the invader cannot persist. Essentially, the invading female ratio allele increases its initial density by “exploiting” males of the resident group while competing for resources with resident females. After some time the density of the resident females reaches zero. The reduced density of females means that the production of males (both resident and invader) is reduced. Consequently, the invading group, once occupying the environment alone, cannot maintain a positive equilibrium density, and a “marriage squeeze” takes the population to extinction.

Given this result, one can envision a stable population where immigration or mutation introduces new alleles over a lengthy time scale. If a new allele has a higher female ratio than the current resident, it will advance. A series of allelic substitutions might increase the female ratio continuously. Our model does not prevent the female ratio from surpassing the threshold defined by Eqs. (4), where the population begins to decline to extinction – recalling Hamilton’s [10] comment on sex linkage and sex-ratio evolution.

### Local Mate Density and Spatial Invasion

#### Invading an open habitat: the critical radius

Equations (1) and (2) assume that densities mix homogeneously, a strong simplification for most organisms. Furthermore, invasion most often has a distinctly spatial character, expanding from one or more foci of introduction [31]. To consider both effects, we assumed a two-dimensional habitat with local mating and random mobility of individuals. This elaborates our model as a reaction-diffusion system [40]. Note, however, that our spatial but deterministic reaction-diffusion equations still maintain an essential (local) “mean-field character” (in the statistical physics sense and terminology) in that all correlation functions are still factorized into products of concentrations [43], [44]. A stochastic, spatial individual-based model or its Langevin-type, stochastic reaction-diffusion analogue (not addressed in this work) may, in principle, lead to different behaviors [45], [46]. For example, the region of persistence in the case of a single-group two-sex population becomes significantly narrower in a stochastic lattice-based model [12].

Successful invasion in spatial environments ordinarily requires that an initial invader cluster have some minimal size for further growth [31], [37], [39], [47]. This criterion may be due to an Allee effect [47] or inherent geometrical constraints on cluster expansion [39]. For systems exhibiting the Allee effect under homogeneous mixing, one can specify this minimal cluster size as the critical radius (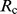) required for spatial invasion. Assuming radially symmetric growth, one expects 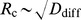, where 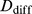 is the diffusion coefficient [47]. For simplicity, we take 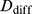 as a constant across all individuals. The first goal of our spatial analysis was to confirm this scaling relationship for the critical radius when a single group is introduced in an open (unoccupied) habitat.

For spatial invasion in an open habitat, individuals diffusing away from the perimeter of the invader cluster encounter mate densities too low for population increase, given the Allee effect (*i.e*., extinction is stable). A small invader cluster can shrink as a result. A cluster size exceeding the critical radius generates interior densities sufficient to drive cluster expansion. The critical radius depends on both density inside the cluster and the diffusion coefficient. Therefore, calculating a critical radius demands specifying initial densities within the circular cluster. We noted that as we chose densities closer to, but exceeding, the Allee threshold of the homogenous-mixing case, the critical radius increased. Therefore, a reasonable (deterministic) choice is the stationary density of the non-spatial model, which we can calculate, given the female ratio and sex-specific mortality rates [see Eq. (S6)].

**Figure 6:**
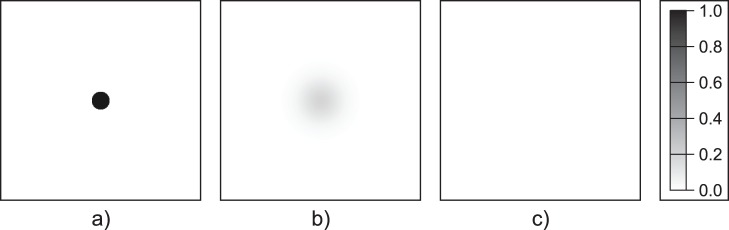
Unsuccessful spatial persistence in the single-group system. Population dynamics in the single-group system (open habitat), where the initial radius is less than the critical radius (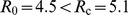). Simulation time: (a) 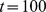, (b) 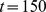, (c) 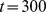. Parameters: 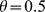, 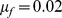, 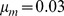, 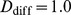.

**Figure 7:**
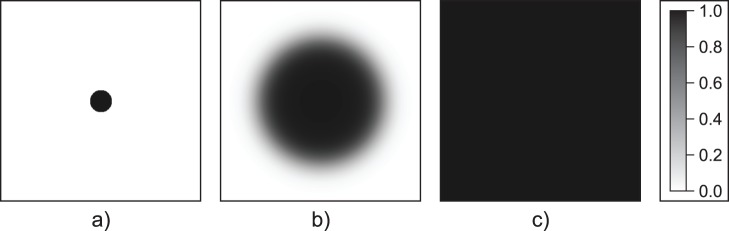
Successful spatial persistence in the single-group system. Population dynamics in the single-group system (open habitat), where the initial radius is greater than the critical radius (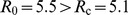). Simulation time: (a) 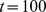, (b) 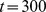, (c) 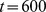. Parameters: 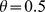, 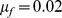, 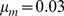, 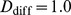.

We found the critical radius by performing a binary search, using the initial interval of 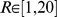. At each step, a simulation runs with a particular initial radius, until all densities at all grid points come to a stationary state (where all time derivatives are less than 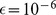). In this final state either all grid points have the positive, stationary densities of the non-spatial model, or all have zero densities. The resolution of the grid (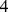 cells/unit distance) and the discretization of a circle on a rectangular grid allow us to measure non-integer radii. Time evolution of a shrinking (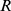 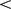 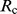) and a successfully growing, invading population (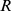 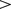 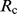) are illustrated in Figures 6 and 7, respectively.

**Figure 8:**
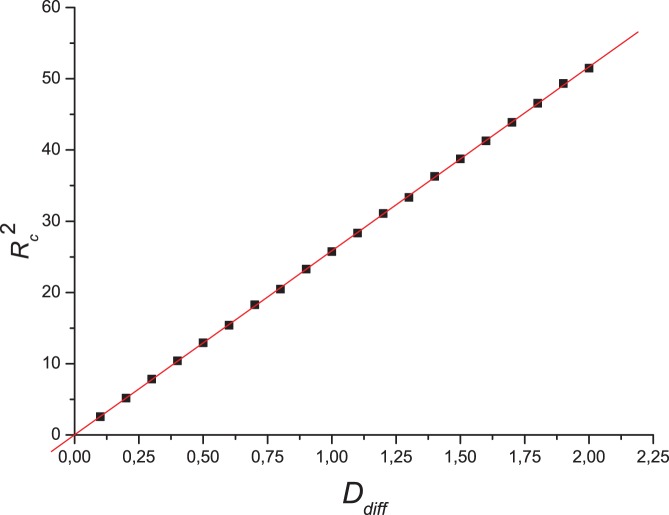
Behavior of the critical radius of the single-group system. Square of the critical radius of a population at self-regulated equilibrium of a single-group system, as a function of the diffusion coefficient (common for both sexes). The line shown is a fitting using minimum least squares, with a correlation value of 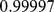. Parameters: 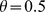, 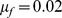, 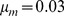.

We obtained the critical radius for various diffusion coefficients, at certain fixed set of parameters [Figure 8]. As anticipated [47], the results confirm that the critical radius is proportional to the square root of the diffusion coefficient.

#### Spatial invasion of a resident population

We extended the between-group competition model to the spatial case with diffusion, using the same grid size, resolution, and diffusion coefficients as we used in the open-habitat model. The goal here is to ascertain if there is a critical radius for successful invasion when invaders can mate with residents in an occupied habitat.

We initiated simulations differently than in the open-habitat case. Here, every grid point was initialized to the stationary density of the resident group. Then, we introduced the invader within a circle of a given radius, at a small density. The simulation ran until all grid points come to a stationary state (where all time derivatives are less than 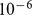).

**Figure 9:**
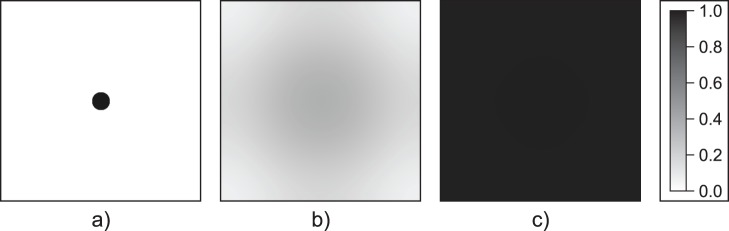
Spatial invasion in the two-group system. Evolution of the invader population density while invading a stable resident population. [Note that the initial radius is less than the critical radius (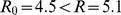) for invasion into an open habitat]. For clarity, only the invaders’ density is shown. Simulation time: (a) 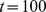, (b) 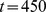, (c) 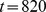. Parameters: 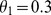, 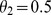, 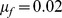, 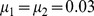, 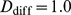.

We found that no matter how small we set the invader density and cluster radius, the result was always identical to the homogeneously mixed case. That is, the allele with the higher female ratio persists, and the ecological impact of the winning female ratio depends on the male mortality rates. Male cultural traits may coexist (type-II fixed point), or both females and males of the lower female-ratio group go extinct (type-I fixed point). Figure 9 shows a scenario where the invader has the same parameters as the open-habitat invasion in Figure 6. However, the result is different, because of the presence of the resident population. The invader can (effectively) exploit the resident population as mates, enabling the invader to spread successfully and eventually exclude the resident.

We understand the absence of a critical radius in the resident-occupied environment by considering cases where even an infinitesimal invader density can completely exclude the resident in the homogenously mixed case. In the worst-case scenario (for the invading allele and culture), we introduce only a small density of invaders at only a single grid point, with a high diffusion rate. Then, diffusion spreads the invader to all grid points, making its density extremely small, but greater than zero. However, this is enough for successful invasion at every grid point, independently of other locations, as we noted in the model with global mixing. If we introduce a greater density of invaders, with slower diffusion, then the invader can quickly overtake the local area before spreading out as a diffusive front. The eventual result will be the same. Hence we conclude that there is no critical radius for invasion with diffusion, if a resident population already occupies the habitat.

## Discussion

Most models of sex ratio evolution, whether analyzed as evolutionarily stable sex allocation [9], [13] or developed with population-genetic detail [4], assume that a parent is related symmetrically to female and male offspring. Hamilton [10] noted that sex-linked inheritance of a gene for sex ratio breaks this symmetry, and extraordinary sex ratios can evolve as a consequence. Frank [48] summarizes effects of asymmetric relatedness to offspring by sex, and cites several studies where this asymmetry is correlated with strongly biased investment in the sexes; see Uyenoyama and Bengtsson [49]. Our results specify how the degree of bias can interact with a between-sex mortality difference to influence the population dynamic consequences of sex ratio evolution.

Tainaka et al. [12] and Nitta et al. [50] developed spatially detailed models to study how sex ratio might affect population persistence. For successful mating, their model requires that at least one fertile individual of each sex occupy a site neighboring an empty site (where the offspring is placed). At the scale of individuals, the dynamics is the simplest generalization of the contact process [51]–[53] that can capture both two-sex reproduction and preemptive competition [30], [31], [38]. Given female and male mortality rates, they find the sex ratio maximizing population density, and note that sex ratios differing too much from this singular value lead to population extinction [12]. Compared to the mean-field result, the extinction effect due to biased sex ratio sharpens in simulation of the stochastic, lattice-based model; the range of sex ratios producing population persistence becomes quite narrow. Since mating pairs form locally, biasing the sex ratio rapidly diminishes the chance that an open site will be neighbored by one individual of each sex. So, demographic stochasticity may lead to extinction once sex ratio is biased, and genetic drift may permit biased sex ratios to evolve even when bias is selectively disfavored [54].

Our study generalizes the model of Tainaka et al. [12] by including between-sex differences in mortality and detailing outcomes of competition between different female ratios. Our model limits expression of sex ratio to the heterogametic sex, so that stronger bias in sex allocation has a competitive advantage. Our results elucidate the ecological effects of interaction among the degree of sex ratio bias and sex-specific mortality for competitive/cultural invasion and demographic stability. In the simplest case, an introduced female allocation and associated cultural trait, male mortality, invades and excludes the resident allele and culture. Complete exclusion requires only that the invaders have the higher female allocation and that their male mortality rate is lower than twice that of the resident males. If the invader’s male mortality rate is large enough to exceed this limit, but the difference in female allocation remains, the resident culture (but not the resident allele) survives and coexists with the invader’s culture.

Our analysis also identified an interesting invasion-to-extinction scenario. A group with the greater female allocation and greater male mortality (compared to the demographically stable resident) cannot invade an empty environment. Yet it invades and excludes the resident, and then goes extinct, because of its high female ratio. Since the invaders can mate with the residents, they effectively exploit the resident group in the early phase of invasion and, when sufficiently numerous, drive the resident extinct. Thereafter, a marriage squeeze leaves the invader declining to extinction. This type of outcome, where sex ratio and an Allee effect can push a population to extinction, may have application in the management of pest populations [16]. Evolutionarily, the demographic consequences of sex ratio bias may favor suppression of sex-ratio distorters [10], and may promote (or be tolerated by) clonal reproduction [55].

The basic two-group two-sex model we considered in this work also allows for some straightforward, yet rich generalizations. In this paper we focused on the scenario where following mating between females and males of different groups, male offspring acquire either cultural trait with probability 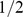. To capture asymmetry in the biparental transmission of the cultural trait in males, our model and the corresponding equations can be generalized to an asymmetric case where male offspring resulting from mating between a female of group 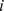 and a male of group 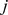 acquire the cultural trait of group 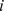 or group 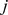 with probability 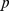 and 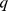, respectively (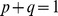). (Vertical cultural-transmission probabilities can, indeed, vary across different combinations of parental phenotypes [25].) While we do not analyze this asymmetric model in detail, we included the corresponding homogeneous mean-field equations and their fixed points in File S1 with the basic findings and note that the qualitative behavior of the system remains the same. In particular, both type-I and type-II fixed points exist, corresponding to full invasion/exclusion and partial invasion/cultural coexistence, respectively. Naturally, for 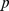 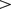 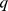 (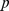 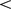 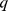) the size of the parameter region with cultural coexistence narrows (widens) and the size of the surviving and coexisting resident culture decreases (increases).

Note: The above generalization (asymmetric cultural transmission in cross-cultural mating) was suggested by an anonymous referee during the review process of this paper.

## Supporting Information

File S1 **Analysis of the Mean-Field Fixed** Points.
(PDF)
