# Supplementary material for: Extraordinary Sex Ratios: Cultural Effects on Ecological Consequences
Source: PLoS One. 2012 Aug 27;7(8):e43364. doi: 10.1371/journal.pone.0043364 (PMC3428370; doi:10.1371/journal.pone.0043364)
Supplement: File S1 — Analysis of the Mean-Field Fixed Points. (PDF) [file pone.0043364.s001.pdf]

Supporting Information: Analysis of the Mean-Field Fixed Points

Extraordinary Sex Ratios: Cultural Effects on Ecological Consequences

F. Molnár Jr., T. Caraco, G. Korniss

Stationary solutions for a single female ratio

When we consider a single female-ratio allele and a single male mortality rate only, the model reduces to the mean-field equations of Tainaka et al. [1]:

$$\begin{aligned} \partial_t F &= \theta\,(1-N)\,FM - \mu_{\mathrm{f}} F \\ \partial_t M &= (1-\theta)\,(1-N)\,FM - \mu_{\mathrm{m}} M \ , \end{aligned} \tag{S1}$$

where the total global density $N = F + M$. For clarity we have dropped subscripts for the resident group. These equations, in general, can have three fixed points. One of these is the equilibrium at extinction:

$$(F^{\mathrm{o}}, M^{\mathrm{o}}) = (0, 0) \tag{S2}$$

To obtain the non-trivial fixed points, we first manipulate the two stationary state equations, Eqs. (S1), to write a simple quadratic equation for the stationary total density,

$$N(1-N) = \frac{\mu_{\mathrm{f}}}{\theta} + \frac{\mu_{\mathrm{m}}}{1-\theta} \ , \tag{S3}$$

yielding solutions

$$N^{\pm} = \frac{1 \pm \sqrt{D}}{2} \tag{S4}$$

with

$$D(\mu_{\mathrm{f}}, \mu_{\mathrm{m}}, \theta) = 1 - 4\left(\frac{\mu_{\mathrm{f}}}{\theta} + \frac{\mu_{\mathrm{m}}}{1-\theta}\right) \ . \tag{S5}$$

Finally, for the non-trivial female and male densities at equilibrium, we have

$$(F^{\pm}, M^{\pm}) = \left(\frac{\mu_{\mathrm{m}}}{1-\theta} \cdot \frac{1}{1-N^{\pm}} \ , \ \frac{\mu_{\mathrm{f}}}{\theta} \cdot \frac{1}{1-N^{\pm}}\right) \ . \tag{S6}$$

For $D \geq 0$ all three fixed points are real. The trivial (zero density) solution [Eq. (S2)] and the "+" solution [Eq. (S6)] are locally stable, separated by an unstable (saddle) fixed point, the "−" solution in Eq. (S6) [the stability of these fixed points can be easily analyzed by linearizing Eqs. (S1)]. For $D < 0$, however, only one biologically meaningful (real) fixed point exists, the zero-density solution [Eq. (S2)], and extinction is always stable.

The biological significance of the structure of the above solutions is two-fold [1]. First, for $D > 0$, the system exhibits the Allee effect. Unless the (initial) population density is sufficiently high ($N(0) > N^{-}$), the population goes extinct. Second, provided that $\sqrt{\mu_{\mathrm{f}}} + \sqrt{\mu_{\mathrm{m}}} < 1/2$, there is a finite interval $\theta_{\mathrm{c1}}(\mu_{\mathrm{f}}, \mu_{\mathrm{m}}) < \theta < \theta_{\mathrm{c2}}(\mu_{\mathrm{f}}, \mu_{\mathrm{m}})$, where $D(\mu_{\mathrm{f}}, \mu_{\mathrm{m}}, \theta) > 0$, i.e., where the population can persist at equilibrium (see Fig. S1). These boundaries, functions of the culturally transmitted mortality rate, are given by:

$$\theta_{\mathrm{c1,2}}(\mu_{\mathrm{f}}, \mu_{\mathrm{m}}) = \frac{(1 + 4\mu_{\mathrm{f}} - 4\mu_{\mathrm{m}}) \pm \sqrt{(1 + 4\mu_{\mathrm{f}} - 4\mu_{\mathrm{m}})^2 - 16\mu_{\mathrm{f}}}}{2} \ . \tag{S7}$$

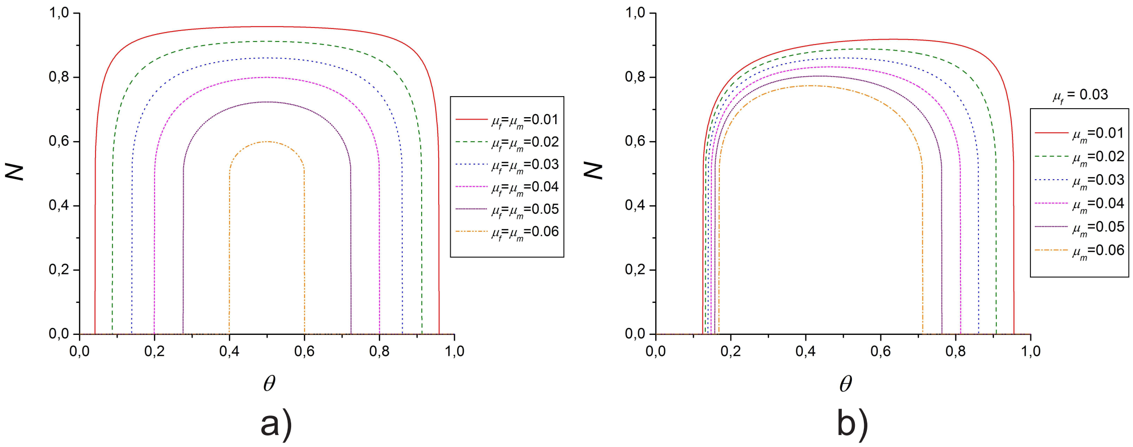

Figure S1. Stationary total population density in the single-allele model [1] as a function of female ratio at birth, for various mortality rates. (a) For identical female and male mortality rates; (b) female and male mortality rates differ.

Between the two critical points, at

$$\theta^* = \frac{1}{1+\sqrt{\mu_\mathrm{m}/\mu_\mathrm{f}}} \,, \tag{S8}$$

total global density exhibits a maximum

$$N^{\max} = N^+(\theta^*) = \frac{1+\sqrt{1-4(\sqrt{\mu_\mathrm{f}}+\sqrt{\mu_\mathrm{m}})^2}}{2} \tag{S9}$$

where the female to male density ratio is $F^*/M^* = \sqrt{\mu_\mathrm{m}/\mu_\mathrm{f}}$.

Stationary solutions for competing groups

The four equations describing the competitive dynamics,

$$\begin{aligned}
\partial_t F_1 &= \theta_1\,(1-N)\,F_1\,(M_1+M_2) - \mu_\mathrm{f} F_1\\
\partial_t M_1 &= (1-N)\left[(1-\theta_1)\,F_1\left(M_1+\frac{M_2}{2}\right)+(1-\theta_2)\,F_2\left(\frac{M_1}{2}\right)\right] - \mu_1 M_1\\
\partial_t F_2 &= \theta_2\,(1-N)\,F_2\,(M_1+M_2) - \mu_\mathrm{f} F_2\\
\partial_t M_2 &= (1-N)\left[(1-\theta_2)\,F_2\left(\frac{M_1}{2}+M_2\right)+(1-\theta_1)\,F_1\left(\frac{M_2}{2}\right)\right] - \mu_2 M_2 \,,
\end{aligned} \tag{S10}$$

are considerably more complex than those of the single female-ratio case. Some of the fixed points, however, directly reflect those of the single-allele case. We obtained the remainder algebraically. Of course, the competitive dynamics has the trivial fixed point where all densities vanish.

Type-I fixed points: genetic and cultural exclusion

The system admits stationary solutions where one allele and the associated cultural trait are excluded, and the other allele has two non-zero fixed points: the "−" solutions in Eq. (S6) are always unstable,

while the "+" solutions are stable if the male mortality rate of the allele with the higher female ratio is less then twice the male mortality rate of the other allele. For example, when allele 1 is excluded, these two fixed points are

$$\begin{aligned}
(F_1^{\pm}, M_1^{\pm}) &= (0\ ,\ 0) \\
(F_2^{\pm}, M_2^{\pm}) &= \left(\frac{\mu_2}{1-\theta_2} \cdot \frac{1}{1-N^{\pm}}\ ,\ \frac{\mu_{\mathrm{f}}}{\theta_2} \cdot \frac{1}{1-N^{\pm}}\right)\ ,
\end{aligned} \tag{S11}$$

where $N^{\pm}$ is given by Eqs. (S4) and (S5) with $\mu_{\mathrm{m}} = \mu_2$ and $\theta = \theta_2$. Further, the "+" solution above is stable provided that $\mu_2/\mu_1 < 2$ and $\theta_2 > \theta_1$. The stability of these fixed points was checked numerically.

Considering the two symmetric cases by interchanging the extant allele/culture with that excluded, we have four fixed points of this type. We refer to stable fixed points of this type [i.e., where the densities of the extant allele are given by the "+" solution in Eqs. (S11)] as type-I fixed points.

Type-II fixed points: cultural coexistence

Next, we obtained fixed points *not* related to those of the single-allele dynamics. Numerical exploration never revealed coexistence of both alleles and both cultural trait values. However, we did find equilibrium populations with a single female-ratio allele and a male cultural dimorphism. Consider, e.g., $F_1 = 0$. The remaining equations for the stationary state then become

$$\begin{aligned}
0 &= (1-N)\,(1-\theta_2)\,F_2\left(\frac{M_1}{2}\right) - \mu_1 M_1 \\
0 &= \theta_2\,(1-N)\,F_2\,(M_1+M_2) - \mu_{\mathrm{f}} F_2 \\
0 &= (1-N)\,(1-\theta_2)\,F_2\left(\frac{M_1}{2} + M_2\right) - \mu_2 M_2\ ,
\end{aligned} \tag{S12}$$

where the overall density is now $N = M_1 + M_2 + F_2$. After some tedious algebra, we again find a simple quadratic equation for the overall density:

$$N(1-N) = \frac{\mu_{\mathrm{f}}}{\theta_2} + \frac{2\mu_1}{1-\theta_2}\ , \tag{S13}$$

which has the solutions

$$\tilde{N}^{\pm} = \frac{1 \pm \sqrt{\tilde{D}}}{2} \tag{S14}$$

with

$$\tilde{D}(\mu_{\mathrm{f}}, \mu_1, \theta_2) = 1 - 4\left(\frac{\mu_{\mathrm{f}}}{\theta_2} + \frac{2\mu_1}{1-\theta_2}\right)\ . \tag{S15}$$

The fixed points follow from Eqs. (S12) after some further elementary manipulations

$$\begin{aligned}
(F_1^{\pm}, M_1^{\pm}) &= \left(0\ ,\ \frac{\mu_2/\mu_1 - 2}{\mu_2/\mu_1 - 1} \cdot \frac{\mu_{\mathrm{f}}}{\theta_2} \cdot \frac{1}{1-\tilde{N}^{\pm}}\right) \\
(F_2^{\pm}, M_2^{\pm}) &= \left(\frac{2\mu_1}{1-\theta_2} \cdot \frac{1}{1-\tilde{N}^{\pm}}\ ,\ \frac{1}{\mu_2/\mu_1 - 1} \cdot \frac{\mu_{\mathrm{f}}}{\theta_2} \cdot \frac{1}{1-\tilde{N}^{\pm}}\right)
\end{aligned} \tag{S16}$$

These fixed-point densities are biologically meaningful (real and positive) if $\tilde{D} > 0$ and $\mu_2/\mu_1 > 2$. The "−" solution above is always unstable, while the "+" solution can be stable if $\theta_2 > \theta_1$ and a number of other necessary conditions are satisfied (described below). We refer to stable fixed points given by the "+" solution in Eqs. (S16) as type-II fixed points of the two-allele system. The necessary conditions

for existence of these stable fixed points are $\sqrt{\mu_\mathrm{f}} + \sqrt{2\mu_1} < 1/2$ [from Eq. (S15)] and $\mu_2/\mu_1 > 2$ [from Eq. (S16)]. In this case, there is a finite range of $\tilde{\theta}_{\mathrm{c}1}(\mu_\mathrm{f},\mu_1) < \theta_2 < \tilde{\theta}_{\mathrm{c}2}(\mu_\mathrm{f},\mu_1)$ where $\tilde{D}(\mu_\mathrm{f},\mu_1,\theta_2) > 0$, so that cultural coexistence persists. The boundaries of this coexistence region are given by:

$$\tilde{\theta}_{\mathrm{c}1,2}(\mu_\mathrm{f},\mu_1) = \frac{(1+4\mu_\mathrm{f}-8\mu_1) \pm \sqrt{(1+4\mu_\mathrm{f}-8\mu_1)^2 - 16\mu_\mathrm{f}}}{2} \, . \tag{S17}$$

Within this regime, the overall population density is maximal at $\theta_2^* = 1/(1+\sqrt{2\mu_1/\mu_\mathrm{f}})$ and the overall female to male density ratio is $F_2^*/(M_1^*+M_2^*) = \sqrt{2\mu_1/\mu_\mathrm{f}}$. The stability of these fixed points was checked numerically. Interestingly, at the stable fixed point in Eq. (S16) the male density ratio is $M_1/M_2 = \mu_2/\mu_1 - 2$; hence the relative abundances of the male cultural trait values do not depend on the female ratio.

Analogously, one can obtain fixed points of the same form as above by choosing $F_2 = 0$ and simply interchanging indices 1 and 2 in all respective expressions. Thus, combined, there are four fixed points of this sort (consisting of both females and males of one group and only males from the other group).

Considering all of the above, we have nine fixed points of Eqs. (S10). Furthermore, a check with Mathematica [2] assures that there are no other fixed points.

Asymmetric cultural transmission in cross-cultural mating

One can generalize the homogeneous mean-field equations (S10) to capture asymmetry in the biparental transmission of the cultural trait in males [3]. Cavalli-Sforza and Feldman [4] point out that vertical cultural-transmission probabilities can vary across different combinations of parental phenotypes. In our model, male offspring resulting from mating of a female of group i with a male of group j acquire the cultural trait of group i or group j with probability p and q, respectively ($p+q=1$). The corresponding equations then read

$$\begin{aligned}
\partial_t F_1 &= \theta_1\,(1-N)\,F_1\,(M_1+M_2) - \mu_\mathrm{f} F_1 \\
\partial_t M_1 &= (1-N)\,[(1-\theta_1)\,F_1\,(M_1+pM_2) + (1-\theta_2)\,qF_2M_1] - \mu_1 M_1 \\
\partial_t F_2 &= \theta_2\,(1-N)\,F_2\,(M_1+M_2) - \mu_\mathrm{f} F_2 \\
\partial_t M_2 &= (1-N)\,[(1-\theta_2)\,F_2\,(pM_1+M_2) + (1-\theta_1)\,qF_1M_2] - \mu_2 M_2 \, .
\end{aligned} \tag{S18}$$

The above equations allow for the same type-I fixed points as their symmetric counterpart Eq. (S11), with no change of the form of the stable density of the surviving group (e.g., in complete invasion/exclusion). The stability domain of this fixed point changes however (see below).

Type-II fixed points, corresponding to cultural coexistence are also possible, given by the solutions of the following equations (e.g., for $F_1{=}0$, $M_1{\neq}0$, $F_2{\neq}0$, $M_2{\neq}0$),

$$\begin{aligned}
0 &= (1-N)\,(1-\theta_2)\,qF_2M_1 - \mu_1 M_1 \\
0 &= \theta_2\,(1-N)\,F_2\,(M_1+M_2) - \mu_\mathrm{f} F_2 \\
0 &= (1-N)\,(1-\theta_2)\,F_2\,(pM_1+M_2) - \mu_2 M_2 \, .
\end{aligned} \tag{S19}$$

The corresponding fixed points then become

$$\begin{aligned}
(F_1^\pm, M_1^\pm) &= \left(0\,,\, \frac{\mu_2/\mu_1 - 1/q}{\mu_2/\mu_1 - 1} \cdot \frac{\mu_\mathrm{f}}{\theta_2} \cdot \frac{1}{1-\tilde{N}^\pm}\right) \\
(F_2^\pm, M_2^\pm) &= \left(\frac{\mu_1/q}{1-\theta_2} \cdot \frac{1}{1-\tilde{N}^\pm}\,,\, \frac{p/q}{\mu_2/\mu_1 - 1} \cdot \frac{\mu_\mathrm{f}}{\theta_2} \cdot \frac{1}{1-\tilde{N}^\pm}\right) \, ,
\end{aligned} \tag{S20}$$

where $p = 1 - q$ and

$$\tilde{N}^{\pm} = \frac{1 \pm \sqrt{\tilde{D}}}{2} \tag{S21}$$

with

$$\tilde{D}(\mu_{\rm f}, \mu_1, \theta_2, q) = 1 - 4\left(\frac{\mu_{\rm f}}{\theta_2} + \frac{\mu_1/q}{1-\theta_2}\right) . \tag{S22}$$

The above equations imply that type-II stable fixed point (S20) can only exist for $\sqrt{\mu_{\rm f}} + \sqrt{\mu_1/q} < 1/2$, $\mu_2/\mu_1 > 1/q$, and $\theta_2 > \theta_1$, provided that $\tilde{\theta}_{\rm c1}(\mu_{\rm f}, \mu_1, q) < \theta_2 < \tilde{\theta}_{\rm c2}(\mu_{\rm f}, \mu_1, q)$,

$$\tilde{\theta}_{\rm c1,2}(\mu_{\rm f}, \mu_1, q) = \frac{(1 + 4\mu_{\rm f} - 4\mu_1/q) \pm \sqrt{(1 + 4\mu_{\rm f} - 4\mu_1/q)^2 - 16\mu_{\rm f}}}{2} . \tag{S23}$$

These conditions define the region where cultural coexistence persists. In this region the ratio of coexisting cultures [male density ratio for fixed point (S20)] is $M_1/M_2 = (q/p)(\mu_2/\mu_1 - 1/q)$.

References

1. Tainaka K, Hayashi T, Yoshimura J (2006) Sustainable sex ratio in lattice populations. Europhys. Lett. 74: 554–559.

2. Wolfram Research, Inc. (2010) Mathematica Version 8.0. Champaign, IL: Wolfram Research, Inc.

3. This generalization was suggested by an anonymous referee during the review process of this paper.

4. Cavalli-Sforza LL, Feldman MW (1981) Cultural transmission and evolution: a quantitative approach. Princeton: Princeton University Press.
